# Effectiveness of preventive dental programs offered to mothers by non-dental professionals to control early childhood dental caries: a review

**DOI:** 10.1186/s12903-019-0862-x

**Published:** 2019-08-02

**Authors:** Ajesh George, Mariana S. Sousa, Ariana C. Kong, Anthony Blinkhorn, Tiffany Patterson Norrie, Jann Foster, Hannah G. Dahlen, Shilpi Ajwani, Maree Johnson

**Affiliations:** 1 Centre for Oral Health Outcomes and Research Translation (COHORT), School of Nursing and Midwifery, Western Sydney University/South Western Sydney Local Health District, Liverpool, NSW 2170 Australia; 2 Ingham Institute for Applied Medical Research, Liverpool, NSW 2170 Australia; 3 Translational Health Research Institute, Campbelltown, NSW 2560 Australia; 4 School of Dentistry, Faculty of Medicine and Health, University of Sydney, Camperdown, 2050 Australia; 5 School of Nursing & Midwifery, Western Sydney University, Penrith, NSW 2560 Australia; 6 Sydney Local Health District Oral Health Services, Sydney Dental Hospital/University of Sydney, Sydney, 2010 Australia; 7 Faculty of Health Sciences, Australian Catholic University, North Sydney, NSW 2060 Australia

**Keywords:** Early childhood, Dental decay, Non-dental professionals

## Abstract

**Background:**

Early childhood caries is a common chronic childhood disease and maternal oral health is a risk factor. Improving the oral health behaviours of pregnant women/young mothers can positively influence the oral health of children and reduce their caries risk. Such preventative strategies have been undertaken by non-dental professionals producing mixed results encompassing various interventions across the perinatal period. However, no comprehensive review of these studies has been undertaken. The aim of this review was to assess the effectiveness of maternal oral health programs undertaken during the antenatal and/or postnatal period by non-dental health professionals to reduce early childhood caries.

**Methods:**

A systematic search of five databases was undertaken using key search terms. Studies were included if they (a) involved quantitative study designs with a control; (b) were published in English; (c) reported on interventions delivered by non-dental professionals (d) delivered the intervention to expectant mothers or mothers with young infants up to 24 months; (e) measured outcomes when the child was under 5 years; (f) measured changes in oral health outcomes of children clinically and oral health behaviours of mothers or children. No restrictions were placed on the study quality and setting.

**Results:**

Nine studies met the inclusion criteria and involved interventions delivered by diverse non-dental professionals across the antenatal (*n* = 1), postnatal (*n* = 6) and perinatal period (*n* = 2). Most studies were of low methodological quality (*n* = 6). The interventions focussed on oral health education (*n* = 8), dental referrals (*n* = 3) and oral health assessments (*n* = 1). Interventions conducted in either the postnatal or antenatal periods showed meaningful improvements in children’s clinical and mother’s behavioural oral health outcomes. The outcomes appear to be sustained when a suite of interventions were used along with referral reminders. There were mixed results from interventions across the perinatal period.

**Conclusions:**

Non-dental professionals can promote maternal oral health by providing oral health education, risk assessment and referrals. Combining these interventions could provide a sustained improvement in oral health outcomes for children although current evidence is weak. More high-quality studies are needed to confirm these findings and determine whether the antenatal and/or postnatal period is best suited to deliver these interventions.

**Electronic supplementary material:**

The online version of this article (10.1186/s12903-019-0862-x) contains supplementary material, which is available to authorized users.

## Background

Early Childhood Caries (ECC) is the single most prevalent chronic childhood disease worldwide despite the fact it can be controlled through targeted changes in diet and oral health behaviours [1, 2]. ECC is characterised by the presence of one or more carious (decayed) teeth in children under 5 years of age [3]. The disease is widespread affecting up to 90% of children worldwide, with higher severity among disadvantaged populations and those from low income countries [4–8]. ECC negatively impacts on children’s lives in both the short and long term, as a result of symptoms associated with untreated ECC such as pain and discomfort. ECC can often lead to problems in everyday activities including eating, sleeping, learning, speech development and growth [1, 4, 9, 10]. Untreated ECC can result in children requiring potentially preventable emergency hospitalisation for caries-related procedures like the removal of carious teeth under general anaesthesia, which can have a psychological impact on both the child and their family [11]. Between 2010/11 to 2013/14, preventable surgeries to treat dental caries constituted 31% of all surgeries conducted among Canadian children between 1 and 5 years of age [12]. In Australia, there were 22,000 cases of preventable hospitalisations due to dental caries reported among Australian children between 1 and 9 years of age in 2011 to 2012 [7].

Although caries aetiology is complex, requiring consideration of environmental, genetic and risk behaviours [13], numerous public health initiatives have been implemented to control and reduce dental caries in children. These initiatives include offering free fluoridated toothpaste [14], pre-school-based brushing [15], mouth rinsing schemes [16], improving access to affordable dental care [17] and school-based fluoride varnish programs [4]. However, many interventions are not implemented before the onset of ECC [18]. For this reason, there has been an increased focus on dental health education and promotion programs for women and new mothers in order to better control the progression of ECC [19]. Though there are changing paradigms on the aetiology of dental caries [13], one mechanism by which children can acquire caries causing bacteria in their first 2 years is through the direct transmission of saliva from mothers; especially if they engage in certain feeding practices, including sharing the same spoon while feeding the baby [20]. Although the transmission of bacteria is virtually impossible to avoid, it proliferates with frequent sugar consumption during the day, night time bottle feeding practices and not brushing their teeth with fluoridated toothpaste when they erupt [21, 22]. Further, educating pregnant women and mothers in minimizing risk behaviours or promoting protective behaviours has been shown to have a positive influence on the oral health of children and reduce their risk of caries progression [23].

Despite government and dental professional initiatives, poor child oral health outcomes continue to persist. This may be attributed to limited access of dental services by mothers and late implementation of preventive dental care interventions aimed at young children. Alongside strategies in this area, there has been growing emphasis in current guidelines on the role that non-dental professionals such as midwives and nurses can play in promoting positive early childhood oral health [24]. Due to the nature of their practice, they are well placed to deliver oral health advice to parents and carers [3, 24]. A review of ECC prevention strategies suggested that paediatric nurses could deliver oral health interventions, and may be an effective means of reducing the prevalence of dental caries [25]. Over the years, a number of dental health prevention programs have been developed and evaluated for the early childhood period utilising non-dental professionals [25, 26]. These studies have produced mixed results and encompassed interventions ranging from oral health education, caries risk assessment and referrals to dental services [27–29]. A comprehensive Cochrane review is presently being undertaken to assess the effectiveness of clinical, health service, policy or oral health promotion interventions that aim to reduce caries in young children by targeting pregnant women and new mothers [30]. Although the Cochrane review findings will be informative, it will not focus on interventions led by non-dental professionals which often can be delivered at different stages of the antenatal and postnatal period [29, 31]. To date no comprehensive review has been undertaken to assess the effectiveness of non-dental professionals administering these oral health programs during either or both the antenatal and postnatal period. Gathering this information will help identify which programs are effective in reducing ECC that can be implemented by non-dental professionals.

## Aims

The aim of this review was to assess the effectiveness of maternal oral health programs undertaken during the antenatal and/or postnatal period by non-dental health professionals to reduce ECC. The term non-dental health professionals refers to all health professionals other than dental professionals. The review has specifically sought to identify the oral health status of children, along with maternal behaviour changes, service utilisation and referrals for dental treatment.

## Methods

The PRISMA statement was used as the basis for reporting the systematic review findings. The protocol for this systematic review was not registered.

### Criteria for inclusion and exclusion

Search strategies were conducted over five databases to include a range of current research: *MEDLINE*, *Science Direct*, *CINAHL*, *ProQuest* and *PubMed*. Literature published up to September 2018 that related to the research aims were included. To reach saturation, key papers were also hand searched to screen for relevant literature. All types of quantitative study designs with a control or comparison group were included in this review. Studies retrospectively assessing outcomes of oral health interventions were also included given they described a comparison group. Studies were included regardless of their methodological quality provided they (a) delivered the intervention to participants who were expectant mothers (antenatal period) or mothers with young infants up to 24 months (postnatal period); (b) outcomes were initially measured when the child was under 5 years; (c) reported on interventions delivered by non-dental health professionals, including oral health promotion, oral health assessments/screening, and referral of participants to dental services, or the intervention was delivered as part of a multidisciplinary team; (d) measured changes in oral health outcomes of children clinically; and (e) measured changes in oral health behaviours of mothers or children.

Studies were excluded according to the following criteria: children were over 24 months of age when the intervention was delivered; parents/caregivers other than mothers were the focus of the intervention and results could not be pooled out separately for mothers; dental professionals administered the intervention to study participants; the non-dental professional was only involved in recruitment; outcomes only measured the mother’s oral health knowledge or behaviour intention; outcomes were not measured postnatally. No restrictions were placed on study setting; however, articles were excluded if they were published in a language other than English. Studies using qualitative methods only, systematic reviews, conference abstracts, dissertations, editorials, commentaries, and non-research articles were also excluded.

### Search strategy

Literature search strategies were individually developed for each database using Medical Subject Headings (MeSH terms), Boolean operators, truncations and a range of alternative terminology and spelling variations. These search strategies utilised keywords derived from the following: *expectant mother, pregnancy, oral health promotion, dental education, preventive dentistry, early intervention, dental caries, prenatal, antenatal* and *postnatal*. The search strategy conducted in each database is found in Table 1.

**Table 1:** Search strategy

Database	Search Terms	Limits
CINAHL	(MH “expectant mothers” OR “prenatal care” OR pregnant OR child+ OR MH “postnatal period+”) AND (MH “oral health” OR MH “oral health promotion” OR “oral health intervention” OR MH “preventive dentistry+” OR MH “dental care” OR “caries prevention” OR “dental referral”)	English
Ovid Medline	(pregnancy OR pregnant women OR prenatal care) AND (oral health/ed. (education) OR “oral health promotion” OR preventative pregnancy OR dental caries OR “early intervention”m.p. or “early intervention (education)”)	English
Pubmed	((“pregnancy”[MeSH Terms] OR “pregnancy”) OR (“pregnant women”[MeSH Terms] OR (“pregnant” AND “women”) OR “pregnant women”) OR (“child, preschool”[MeSH Terms] OR (“child” AND “preschool”) OR “preschool child” OR (“child” AND “preschool”) OR “child, preschool”) OR (“child”[MeSH Terms] OR “child”) OR “early intervention (education)” OR antenatal OR postnatal) AND ((“oral health”[MeSH Terms] OR (“oral” AND “health”) OR “oral health”) AND “oral health promotion” OR (preventative AND (“dentistry”[MeSH Terms] OR “dentistry”)) OR ((“dental caries”[MeSH Terms] OR (“dental” AND “caries”) OR “dental caries”))	English
Science Direct	(pregnancy OR prenatal OR “pregnant women” OR preschool OR child OR “early intervention”) AND (“oral health education” OR “oral health promotion” OR “preventative dentistry” OR “dental caries prevention” OR “oral health intervention”)	English
ProQuest	(pregnancy OR pregnant women OR prenatal OR antenatal OR postnatal OR early intervention) AND (oral health promotion OR oral health screening OR oral health education OR oral health intervention OR caries prevention OR dental visit OR preventative dentistry OR dental education OR dental caries)	English

### Selection process

**Fig. 1:**
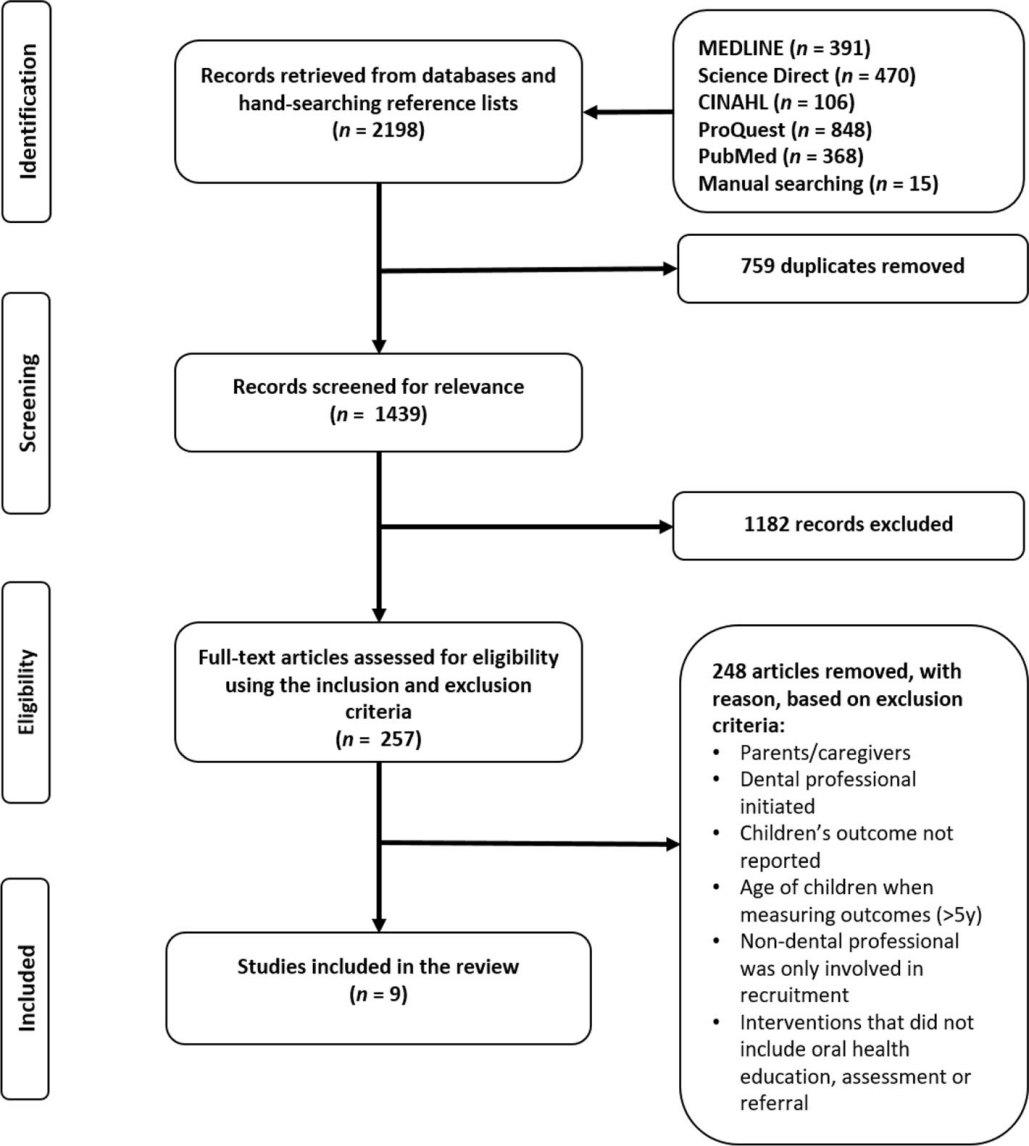
PRISMA diagram of study selection

The search retrieved 2184 records from the five databases and two independent reviewers (MSS, TPN) extracted 15 further articles from the reference lists of key papers. Duplicates were removed and 1439 papers were screened by the two reviewers for relevance to the research aims, where a further 1182 records were excluded. Three reviewers (MSS, ACK, TPN) assessed the full text of 257 papers based on inclusion and exclusion criteria. Disagreements were resolved through discussion; however, a fourth reviewer (AG) was also consulted to assist in reaching consensus. Study authors were contacted to clarify details of the intervention if criterion eligibility was unclear. Where there was no response from the corresponding author after 1 week, the second author was subsequently contacted for clarification (Fig. 1).

### Data extraction process

Data were extracted from each article independently and included study details, aims, design, population demographics, type of non-dental health professionals who delivered the intervention, type and description of intervention, and outcome measures, all of which were described with the time of intervention delivery (i.e. antenatal, postnatal or both periods) (Additional file 1). If the methods or results of papers were inadequately described, we referred to literature cited within the study for elaboration and contacted the study authors for further clarification. Discrepancies in extracted data were resolved through discussion, and data was collated into a single summary table (Additional file 1).

### Outcomes and prioritisation

The oral health outcomes of children, as defined by prevalence of ECC or decayed, missing or filled surfaces (dmfs) and teeth (dmft), were the primary outcome measures to determine the clinical effectiveness of the intervention on improving their oral health. Oral health behaviours were the secondary outcome measure and included variables such as oral health knowledge, practice and dental service uptake as they can assist in predicting future dental outcomes [32].

### Quality of individual studies reviewed

The quality of each study was assessed independently (MSS, ACK) using the National Institute of Health (NIH) study quality assessment tools for systematic evidence reviews and clinical practice guidelines [33] (see Additional file 2). The NIH study quality assessment tool was chosen as it enables a range of quantitative study designs to be evaluated. The quality of each study was expressed by the number of criteria met using ‘1’ (Strong), ‘2’ (Moderate) or ‘3’ (Weak) if they scored 80–100%, 60–79 and < 60% respectively.

## Results

### Characteristics of the included studies – design, setting and demographics

A total of nine primary research studies met the inclusion criteria: one reporting antenatal intervention only [29], six reporting postnatal interventions only [28, 31, 34–37] and two reporting combined antenatal and postnatal interventions [27, 38] (Additional file 1). Our review included five randomized controlled trials (RCTs) [27, 28, 34–36] (two of them being cluster RCTs) [27, 28], three quasi-experimental studies with a control or comparison group [31, 37, 38] and one retrospective chart review [29]. Studies were conducted in five different countries: two in Canada [31, 36], three in the United States [29, 35, 38], two in Brazil [27, 34], one in Ireland [37], and one in Iran [28].

Overall, demographics of the participants (both mothers and children) were poorly described in the studies. The mean age of the mothers was only mentioned in two studies and ranged from 25.7 years [34] to 26.4 years [27] at delivery. Five of the studies provided information about race/ethnicity, which included Latina [35], white [27], Punjabi-speaking South Asian [36, 39] and Vietnamese women [31]. Only four provided information on education level [27, 28, 37, 38] and five on socioeconomic status of participants [27–29, 37, 38]. Mostly, studies included participants residing in areas of high social deprivation, or from high-risk, impoverished, and socioeconomic-challenged communities. The mean age of participating children were only described in four studies and ranged from 11 months [36] to 28 months [37], although the follow-up period ranged from 0 to 7 years. The studies included diverse samples of participants and none of the samples had similar characteristics.

### Methodological quality of the papers

Overall the aims, design, population and settings, intervention and data collection methods were poorly described (Additional files 1 and 3) with a total of six studies being classified as weak.

### Type of non-dental health professionals who provided the intervention

Interventions were provided by different groups of non-dental professionals including health counsellors – local South Asian lay women [36, 39] and a lay Vietnamese woman [31] – community based nurses [37], midwives [35], healthcare workers – physicians, nurses and administrative staff [27] – field workers [34], general vaccination health staff [28], outreach coordinator – a health department employee [38] – and multidisciplinary team formed by nurses, obstetricians, social workers, nutritionists, oral and maxillofacial surgeons and support staff [29]. The majority of studies described these professionals receiving trainings/introductory workshops; nonetheless, only five provided details of the trainer’s background, which included nutritionists [27, 34] and dentists [28, 36, 39].

### Interventions

The intervention methods varied across studies and included (i) oral health education, (ii) oral health assessment/screening and (iii) referrals of participants to dental services. Provision of oral health education by a non-dental health professional was the focus of all the studies. Three also included referrals for dental care by non-dental health professionals – obstetricians [29], community-based nurses [37] and outreach coordinator (OHSC) who was a health department employee [38] – and one involving dental screening initiated by a multidisciplinary team [29].

Oral health education encompassed verbal oral health advice and information such as discussing that dental care is safe during pregnancy [28, 29], one-to-one counselling sessions [27, 31, 38], motivational interviewing [36, 39], home visits for one-to-one preventive advice [34, 37, 38], as well as follow-up phone calls to provide support and to coach mothers [31], to reinforce and maintain behavioural changes [36, 39], and as reminders of oral health instructions [28] and child’s dental appointment [35]. It also involved written oral health promotion materials such as postcards [36, 39], pamphlets [27, 28, 36, 39], brochures [35], leaflets [34], posters displayed in clinics [27], letters to remind parents about a child’s dental appointment [35] and a toolkit containing educational material [38]. Some visual tools were also used in interventions and consisted of educational videos [36, 39] and DVDs [35]. Finally, in some interventions, community-wide initiatives (video, written information, window displays and brochures) were also employed [31] as well as the distribution of ‘goody bags’ that included items such as an adult or infant toothbrush, toothpaste, training cups, finger cots and table mats [31, 35, 37].

The screening/assessment component of the intervention described by Larsen et al. [29] included (but was not limited to) prompting pregnant women regarding ‘current oral health problems, previous dental problems, and the availability of a dental provider’*.* Those identified as having a ‘current oral health problem’ or ‘not having a dental visit in the past six months’ were referred to a dentist, preferably before 20 weeks gestation. The other two studies that also had a referral component involved distribution of dental registration vouchers by nurses [37] and referral by the OHSC during home visits [38].

The exact point in time during the antenatal period, in which the intervention was provided, was not specified by the authors [29]. The postnatal interventions occurred sometime between immediate (0–5 days) and 24 months post-partum [36, 39]. Interventions that took place in both antenatal and postnatal periods occurred from second trimester of pregnancy to within 2 months of delivery [27].

### Measurements

Eight out of the nine studies measured clinical outcomes in children (i.e. dental health status) using proxies such as presence of dental caries/decayed teeth/cavitation [27, 29, 35], extractions [29], decayed surfaces [28, 31, 34, 36, 38], enamel caries [28], and child’s caries risk [35]. In addition to clinical outcomes, six assessed mother’s behaviours including service uptake, beliefs, and dietary, hygiene and parenting practices [27, 29, 31, 34–36]; one children’s dietary behaviour [36]; one mother’s knowledge/awareness of oral health; one mother’s experiences with the intervention [27] and one assessed mother’s perceptions of the effectiveness of the intervention [28]. One study assessed only behavioural outcome through service uptake by preschool children [37].

## Findings

### Antenatal period

Using a retrospective chart review, Larsen et al. [29] investigated the efficacy of an antenatal intervention involving oral health education, referral and screening delivered by a multidisciplinary team (involving obstetricians, nurses, social workers, a nutritionist, oral and maxillofacial surgeons, dentists and support staff), in addition to dental evaluation and consultation by dental professionals to pregnant women. The authors reported that children of mothers who received the intervention had significant clinical outcomes such as less dental caries (*p* = 0.019), fewer extractions (*p* < 0.021) and number of teeth with caries at 2–3 years of age (*p* < 0.001) compared with children of mothers who did not participate in the intervention. Oral health service uptake was also increased overtime following the intervention suggesting its effectiveness in improving oral health of young children.

### Postnatal period

Interventions conducted in the postnatal period also showed meaningful improvements in children’s clinical and mother’s behavioural outcomes. Clinical improvements went from fewer decayed surfaces (*p =* 0.03) [34] and lower enamel caries (de) increment (*p <* 0.05) [28] in the short-term (up to 1 year after the intervention) to fewer decayed surfaces measured as defs (*p <* 0.005) [31] and fewer new carious lesions (*p <* 0.01; [36] and *p <* 0.02) [39]; sustained over the 1-year [36] and follow-up periods [31], confirming the protective effect of oral health education interventions. Due to the high rates of no-show in follow-up assessments, clinical evaluation of the oral health education (using DVDs) described by Hallas et al. [35] was compromised. Baseline data however, highlighted mother’s lack of oral health knowledge, particularly awareness of vertical transmission of *S. mutans* during immediate postpartum.

Behaviourally, changes in mother’s parenting practices such as less use of sleep-time and daytime bottles (*p* < 0.005) [31], dietary practices including duration of exclusive breastfeeding (*p* = 0.000) and introduction of sugar (sugar cane and honey in fruits, milk and porridge etc) (*p =* 0.005) [34] were also significant and contributed to improved oral health outcomes in children. Despite these positive outcomes, the results reported by Feldens et al. [34] were short-term only. Service uptake was also significantly increased for 0–2 year old children, 5 months after a combination of oral health education and referral to dental services intervention but showed no equivalent effect for the 3–5 year old group [37]. Conversely, the oral health education only intervention conducted by Weinstein et al. [36, 39] showed no difference in service uptake between intervention and control groups after 1-year follow-up (around 1–2 years of age) [36].

### Both antenatal and postnatal periods

Interventions offered in both pregnancy periods and postnatal had mixed results. The combined oral health education and referral intervention described by Milgrom et al. [38] showed significant clinical outcomes with reduction in the mean number of teeth with decay (*p* = 0.04) in children up to 2 years living in rural areas. Nonetheless, this finding was primarily attributed to the dental care component of the intervention with the authors acknowledging that non-dental health professionals played a minor role in referring and providing education at home visits. In addition, the study by Chaffee et al. [27] provided oral health education as an intervention during both periods, showed no significant reduction when compared with the control groups. Feldens et al.’s [34] study was similar to the intervention by Chaffee et al. [27] which focussed on nutrition. Physicians and nurses were trained in infant complementary feeding by a nutritionist to incorporate into maternal consultations [27]. However, the weakness of Chaffee et al.’s study was that the number of times women received counselling was not monitored and the accuracy and consistency of the messages relayed to mothers were unknown [27].

## Discussion

The antenatal and postnatal periods are critical moments for key health behavioural changes that impact both the mother and infant. It is a time when predominantly healthy women have considerable contact with health services on a regular basis and will receive important information from health professionals that may affect their or their unborn or newborn child’s health. This is particularly important in relation to the reduction of ECC. We reviewed nine research studies [27–29, 31, 34–38] that included non-dental health professionals or multidisciplinary teams and lay persons to provide some form of preventive dental care advice. Dental services are known to be poorly utilised by women during pregnancy [19] and therefore other health professionals are key to delivering messages relating to oral health during this time. The studies which met the selection criteria provided some direction for others to follow when developing future interventions by non-dental professionals to improve the oral health of pregnant women and their newborn children.

From the studies reviewed, the most appropriate non-dental professional to deliver oral health messages relating to ECC varied from obstetricians [29] to lay people with specific training [31]. However, improvements in clinical and behavioural oral health outcomes were observed irrespective of the person delivering the intervention. Given that oral health advice to clients is often not offered by most non-dental health professionals, the need for specific oral health training for them as part of any intervention especially those involving assessment and referral is essential [40–43]. Oral health training programs have been provided to midwives with positive outcomes for pregnant women [42, 44]. For particular cultural groups, however, the additional use of key lay people with specific training in oral health using tailored screening tools or questions could be advantageous, and potentially cost effective. [44–46].

The types of interventions provided to address ECC varied considerably and focused predominantly on oral health education [28, 29, 31, 35–39] with two studies focusing on nutrition [29, 34] and only one study involving oral health assessment [29]. This could be because undertaking such an intervention requires adequate oral health knowledge and training which is known to be lacking among non-dental professionals (40–43). Time constraints among non-dental professionals in addition to short term oral health programs that do not follow the children’s oral health over time could be other factors why many studies have not included an oral health assessment as part of the intervention [42]. Nevertheless, it appears that comprehensive interventions that include providing information, an oral health toolkit, and face-to-face counselling sessions (either at a clinic or community health centre, including a dental referral and a home visit) can reduce child dental caries and result in children being up to 1.5 times more likely to be caries free [38]. Improved oral health outcomes appear to be sustained over a longer period where the suite of interventions also involved reinforcement through referral or follow up reminders [28, 29, 31, 34, 36–38].

Although no major clinical effect in terms of caries reduction was associated with the combination of counselling and the distribution of posters and pamphlets in the study conducted by Chaffee et al. [27], there appears to be a more protective effect among mothers who were more connected to their health centre. Similarly, studies which involved a combination of face-to-face education or counselling, provision of pamphlets and follow-up visits reported significant improvement in oral health behaviours including increased access to dental services [37] and improved feeding [31] or dietary practices [34]. The cost effectiveness of the less comprehensive, versus the more extensive programs requires further evaluation [27, 34].

Multimedia delivery of education was used in most studies as part of the combined intervention [27, 28, 31, 35–38] with women receiving a DVD [35] or viewing a video providing oral health education as well as written material [31, 36]. Although the use of pamphlets in the study by Mohebbi et al. (2009) [28] was not found to be effective in reducing caries their perception of the pamphlet’s usefulness appeared to be moderated by whether they also received verbal education. The effectiveness of a DVD-only intervention was difficult to determine due to the low retention rate [35]. However, this DVD approach is likely to be a low-cost intervention although no study provided any economic evaluation of their intervention.

Oral hygiene kits were part of the intervention in four studies [31, 35, 37, 38]. The composition of these kits varied from educational material [38], feeding cups [31, 37], tooth brushes and toothpaste [31, 35, 37]. The inclusion of these kits may be more relevant in populations from lower socioeconomic groups or cultural groups where some oral health practices are uncommon. For example a recent study involving Aboriginal Health Workers [47] has shown that the supply of free toothbrushes, toothpastes and feeding cups is critical in improving the oral health of Aboriginal preschool children in Australia.

Lastly due to the paucity of studies focusing on both antenatal and postnatal oral health interventions (especially antenatal), it is difficult to identify the period where interventions for ECC are most effective. Based on the limited evidence it appears that providing oral health education, assessment and referrals during the antenatal period could improve the oral health of children (29). This is not surprising as such interventions can significantly improve the oral health knowledge and oral health outcomes of pregnant women [48] which in turn, could influence early childhood oral health outcomes [21]. What is not clear is whether reinforcement of such interventions in the postnatal period as well will have greater impact on the oral health of children. More high-quality studies are needed across both time periods to confirm this and determine the scope of practice of non-dental professionals in this area.

## Limitations

Almost all studies meeting the inclusion criteria were from upper middle to high -income countries with established healthcare systems. Part of this may be attributed to the bias in only selecting articles available in English. This limits the applicability of findings in different settings and further research in low-income countries is warranted. The methodological quality of the included studies was also rated as generally low although three studies used either a simple randomised or cluster randomised controlled design which is likely to deliver sufficient evidence of intervention effectiveness. Clearly there is a need to improve the scientific rigour of the research undertaken in this field of health promotion.

## Conclusion

Health professionals outside of dentistry can play a key role in promoting maternal oral health. With adequate training these professionals can expand their scope of practice to provide oral health education, risk assessments and dental referrals. Combining such interventions could provide a sustained improvement in oral health outcomes of children although the quality of evidence is weak. More robust studies are needed to confirm these findings and to determine whether the antenatal and/or postnatal period is best suited to deliver these interventions.

## Additional files

Additional file 1: Summary Table of Data Extracted. Details of studies included in the review. (DOCX 44 kb)

Additional file 2: National Health, Lung, and Blood Institute (NIH) Quality Assessment Tool. Tool used to assess the quality of studies included in the review. (DOCX 17 kb)

Additional file 3: Quality Assessment of Included Studies. Results of quality assessment using NIH tool. (DOCX 13 kb)

## Data Availability

All data generated or analysed during this study are included in this published article and in the supplementary files for transparency.

## Abbreviations

CI: Confidence interval
CSA: Central Services Agency, an organisation responsible for providing support services to health and social work agencies in Ireland;
de: enamel caries
dmfs: decayed, missing or filled surfaces
dmft: decayed, missing or filled teeth
DS or ds: Dental surface
Dt: Decayed teeth
ECC: Early Childhood Caries
GDPs: General Dental Practitioner
HE: Health education
Hr: Hour(s)
MeSH: Medical Subject Headings
MI: Motivational interviewing
Min: Minute(s)
mo: month(s)
N/S: Not specified
NIH: National Institute of Health
SD: Standard deviation
S-ECC: Severe early childhood caries
T1/T2: Time-point 1/Time-point 2
Wks: Weeks
Y: Year(s)
