# Supplementary material for: Effectiveness of preventive dental programs offered to mothers by non-dental professionals to control early childhood dental caries: a review
Source: BMC Oral Health. 2019 Aug 2;19:172. doi: 10.1186/s12903-019-0862-x (PMC6679429; doi:10.1186/s12903-019-0862-x)
Supplement: Supplementary file 1 — Summary Table of Data Extracted. Details of studies included in the review. (DOCX 44 kb) [file 12903_2019_862_MOESM1_ESM.docx]

**Additional File 1 Summary Table of Data Extracted**

| **Author (Year)**  **Location** | **Study Design/**  **Study Aims** | **Participants**  **(Sample size and characteristics)** | **Non-dental health professional** | **Description of intervention** | **Outcome measures** | **Findings** | **Quality (1=Strong; 2=Moderate; 3=Weak)** |
| --- | --- | --- | --- | --- | --- | --- | --- |
| **ANTENATAL PERIOD** | | | | | | | |
| **Larsen et al. (2016) (**[**2**](#_ENREF_27)**9)**  **United States** | **Retrospective chart review**  Evaluate if prenatal counseling and care for pregnant mothers promotes oral health in children. | 91 mother-baby dyads  ***Intervention:*** n=42  ***Comparison:*** n=49  ***Mothers***  Multicultural, low-socioeconomic community  ***Children***  Intervention group  7% 1yr  38% 2yr  55% 3yr  Comparison group  10% 0-1yr  24% 2yr  39% 3yr  27% 4yr | Obstetricians referred patients to PCAP.*  Multidisciplinary team provided care:   - Nurses - Obstetricians - Social workers - Nutritionists - Oral and maxillofacial surgeons - Dental support staff | ***Intervention group***  Oral health education   - The care provider delivered oral health education.   Dental screening   - Prenatal care provider performed a woman’s oral health care needs assessments during first visit (interviewed about current/previous dental problems and availability of a dental provider).   Dental referral   - Referral was made (preferably before 20 wks gestation) for dental evaluation and consultation if an oral health problem was identified, or last dental visit was >6 months.   ***Comparison group***  Did not attend PCAP or received dental evaluation/ consultation. Received usual care. | ***Clinical - dental health status***   - Prevalence of children with/without caries and without extraction of four teeth (DEFG) - Average number of teeth with caries - Measured annually (2010-2013)   ***Behaviour – Service uptake***   - Prevalence of mother-child dyads who returned for preventive and treatment visits | ***Clinical***   - Overall, children of PCAP mothers had fewer dental caries than the no PCAP group (31% vs 61%, p=0.019) and fewer extractions (7% vs 31%, p=0.021). - No difference between the 2 groups for average number of caries at 2 y. - Caries were higher at 3 y, 2-3 y and 2-4 y (p<0.001).for children whose mother did not participate in the PCAP | 3 |
| **POSTNATAL PERIOD** | | | | | | | |
| **Harrison et al. (2003) (31)**  **Canada** | **Quasi-experimental with comparison group**  To design, implement and evaluate a culturally sensitive oral health promotion program to improve dental health in Vietnamese preschool children in Canada. | *Not clearly specified*  ***Baseline:*** n=112 Vietnamese mothers with >1 counselling session  ***Follow-up:*** n=66 Vietnamese mother-infant dyads (12-60mo)  ***Comparison****:* Vietnamese children from a neighbouring municipality  ***Children – mean age (SD)***  Baseline group  25.3mo (6.2)  Comparison group  22.7mo (5.8) | Community dental health worker (CDHW)*:   - Health counsellor - Lay Vietnamese women | ***Intervention group***  Oral health education - Counselling   - CDHW provided one-to-one counselling at each recommended immunization schedule (at 2, 4, 6, 12 and 18mo). - Discussed healthy oral hygiene and feeding practices for baby. - Oral health promotion kits provided for infant at each visit. - CDHW routinely made follow-up phone calls for support and coaching to mothers.   Oral health education – Community-wide initiatives   - Videos and articles were disseminated through community-wide initiatives - Other activities included: child dental health booths at local festivals, window displays near bus stops and child oral care brochure for nurses.   ***Comparison group***  Did not receive dental education. | ***Clinical - dental health status***   - Visible evidence of a cavity involving the dentine of a tooth was considered carious (defs) - Visual examinations by project dentist in children over 18mo - Baseline measure (1994) with 4 follow-ups over 7y (1996, 1998, 1999, 2001)   ***Behaviour –Practices, awareness/knowledge***   - Brief baseline and follow-up questionnaires to determine parenting practices, awareness of community outreach activities and suggestions for improving oral health of children. - CDHW collected responses during a brief interview. | ***Clinical***   - Children whose mothers attended >1 counselling session had significantly fewer decayed surfaces compared to children at similar age at baseline (*p*<0.005).   ***Behaviour***   - Mothers with >1 counselling session reported significantly less use of sleep-time and daytime bottles (infant feeding and comforting practices) for children 12-60 mon of age (*p*<0.005). - Results suggest one-to-one counselling with regular follow-up provided by a lay person of similar background/culture to the participants is an effective way to facilitate adoption of healthy behaviours and to improve oral health of children. | 3 |
| **Weinstein et al. (2004) (**[**3**](#_ENREF_30)**6)**  **Canada** | **Randomised controlled trial**  To compare motivational interviewing (MI) with a traditional health education (HE) approach to the prevention of caries in children at high risk of developing dental caries. | 240 mother-infant dyads from a Punjabi-speaking South Asian community  ***Intervention:*** N/S  ***Comparison:*** N/S  ***Children – mean age (range)***  Intervention  11mo (6-18mo)  Comparison  12mo (6-18mo) | Counsellors   - 3 local South Asian women - Trained by the authors | ***Intervention group***  Oral health education – Counselling session (MI)   - Initial visit (45 min): Establish rapport and needs, present and discuss menu options, use strategies that reinforce behavioural change. - 6 phone call and 2 postcard reminders: Cue and reinforce behavioural change and solve any problems.   Oral health education – Pamphlet and video   - Pamphlet designed by health staff and reviewed by an author - 11-min video (5 languages). - Both were modified to include dietary and non-dietary ECC-preventive strategies appropriate to South Asian community and encouraged parents to take their children to a community organization for fluoride varnish application.   ***Comparison group***  Oral health education – Pamphlet and video  Same as per intervention group | ***Clinical - dental health status***   - Visual examination using the modified Radike criteria (decayed or filled surfaces) - Assessed by calibrated dental professional examiners - Measured at baseline and Year 1 follow-up   ***Behaviour – Service uptake***   - Average number of visits for fluoride varnish applications | ***Clinical***   - Children in the MI group had 0.71 new carious lesions (SD 2.8, range=0-25) compared to those in the control group that had 1.91 (SD 4.8, range 0-25) new carious lesions (*p*<0.01). - This suggests that MI counselling has a greater clinical meaningful effect on ECC than traditional HE.   ***Behaviour***   - No differences in average number of dental visits for fluoride varnish applications were reported between groups. | 3 |
| **Yuan et al. (2007) (37)**  **Ireland** | **Quasi-experimental non-equivalent two group comparison**  To evaluate the effectiveness of a community-based program to promote dental registration and access to dental services for preschool children residing in areas of high social deprivation using monthly registration data provided by the Central Services Agency (CSA). | 23 wards  ***Intervention:*** n=9  (3 urban and 6 rural)  ***Comparison:*** n=14 (6 urban and 8 rural)  ***Mothers of newborn babies***  Recruited based on socioeconomic status and whether they were living in rural/urban regions | 12 community-based nurses (health visitors)  44 General  Dental Practitioner (GDPs)   - Provided preventative advice   Both received an introductory workshop* | ***Intervention group***  Oral health education – Home visits   - Health visitors provided oral health education to empower mothers to register after eruption of 1st deciduous tooth and to re-register for continuous dental care (after 15mo). - Distributed oral hygiene products   Dental referral   - The health visitor distributed dental registration vouchers and list of dentists in the areas.   Oral health education – GDP appointment   - One-to-one preventive advice provided by GDP on how to care for baby’s teeth (how to reduce the need for pain-only attendance and how to maintain child’s registration with the practice) - Mothers exchanged the vouchers for motivational materials from the participating dental practices.   ***Comparison group***  Was not provided intervention as described above. Received usual care. | ***Behaviour – Service uptake***   - Dental registration rates for preschool children (0-2y and 3-5y) - Obtained from the CSA to evaluate the effectiveness of the program - Measured rates at baseline, i.e. 6mo before program (November 2001-April 2002), T1 during the program (May 2002-April 2004), and T2 5mo after program completion (May 2004-September 2004). | ***Behaviour***   - T1: The rate of change in registration for children 0-2yr in the intervention wards was significantly greater compared with controls (t [DF:21]=4.26: p<0.001). - T2: Statistically significant service uptake at T2 and increase in registration rate was seen for 0-2yr old children in the intervention wards compared with controls (t [df: 21]=3.33: P=0.003). - There were no equivalent effects for the 3-5yr group.   The CSA data indicated that the community-based dental registration program had increased access to dental care for 0-2yr children in the intervention wards and had the potential to enable mothers to maintain dental registration for their 0-2yr olds. | 3 |
| **Feldens et al. (2007) (**[**3**](#_ENREF_33)**4)**  **Brazil** | **Randomised controlled trial**  To examine the effectiveness of home visits for advising mothers about breast feeding and weaning on children’s feeding practices and general health in reducing ECC in the first year of life. | 500 mother-child dyads.  ***Intervention:*** n=200  ***Comparison:*** n=300  ***Mothers***   - Mean age at child’s birth 25.7y - Mean schooling 6.8y - 34.3% had a paid work - 72% low income (up to 3 minimum wage)   ***Children***   - 56.4% boys - 63.7% were 2nd born or greater child | 12 fieldworkers   - Delivered intervention - Received 8hr of theoretical training and 8hr of practical training - Training based on a detailed adviser guide prepared for the study and calibrated against the advice by a paediatric nutritionist | ***Intervention group***  Oral health education – Home visits   - Verbal dietary, healthy breast-feeding and weaning advice (based on WHO recommendations) was given to mothers by fieldworkers during home visit (designed to tackle cariogenic feeding practices and sweet intake) - Mothers were also given a leaflet (as advice and as a reminder) - Home visits occurred within 10 days after birth; monthly up to 6mo; and at 8, 10 and 12mo. - Received routine assistance by paediatrician   ***Control group***  Dietary advice related to oral health by fieldworker after the 12mo research assessment. Received routine assistance by paediatrician | ***Clinical - dental health status***   - Visual examination by a paediatric dentist of each erupted teeth and ds – presence of caries in any ds was considered as ECC. - Number of teeth and DS calculated for each child. - Measured within 1mo after the 12mo home visit.   ***Behaviour –Practices***   - Dietary behaviour (onset, duration and frequency of feeding practices) at 6 and 12mo. - Collected through face-to-face interviews conducted by fieldworkers. - Interview used standardised forms and interview guide. | ***Clinical***   - Intervention group had significantly lower rates of ECC compared to the comparison group at 1 yr (10.2% (16/157) vs 18.3% (40/219).Likelihood of caries at the age of 1yr was 48% lower in the intervention group (OR 0.52; CI 95% 0.27-0.97) compared to the control group after adjustment for the confounding effect of number of teeth. The mean number of mean ds were also lower in the intervention group (0.37 vs 0.63,*)* compared to the control group. The difference in average number of ds between the 2 groups was statistically significant (*p=0.03).*   ***Behaviour***   - Duration of exclusive breastfeeding was significantly longer in the intervention group (*p*=0.000) and age at which sugar was introduced was significantly higher (*p*=0.005) compared to the control group.   Although, a relevant proportion of children of the intervention group also had ECC, in the short-term, home visits for dietary advice appear to help reducing ECC. | 1 |
| **Mohebbi et al. (2009) (**[**2**](#_ENREF_26)**8)**  **Iran** | **Cluster randomised controlled trial**  To evaluate the impact of a 6-mon educational intervention on ECC (dentinal and enamel caries) in 12 to 15-mon olds. | 242 mother-child dyads  ***Group A:*** n= 77  ***Group B:*** n= 85  ***Control:*** n=80  Health centres: n=18 (6 in each group)  ***Mothers***   - Income and education levels poorly defined   ***Children***   - 50% male - Mean age 12.3mo (SD 0.4) (range: 12-15mo) | 36 general (vaccination) health staff   - Two staff ‘Health staff’ recruited from each centre - Two members of the health staff received 1-2hr training from a dentist who supervised the intervention. | ***Intervention groups***  Group A: Oral health education   - Pamphlet on caries prevention (in Persian) distributed at baseline. - Verbal oral health instructions (5 min) delivered at baseline. - Reminder oral health instructions (twice at 2mo intervals) over the phone.   Group B: Oral health education   - Same pamphlet on caries prevention (in Persian) at baseline.   ***Control group***  No dental information during 6mo period. After the intervention, mothers received the same pamphlet on caries prevention from the vaccination health staff. | ***Clinical - dental health status***   - Number needed to treat (NNT) - the smaller the NNT, the grater the effectiveness of the intervention. - Assessed difference in caries increments: (i) number of dt and de and (ii) percentages of children developing new dt and de. - Conducted by dentists - Measured at baseline and 6mo follow-up   ***Mother’s perception of the effectiveness of the intervention***   - Brief interviews conducted by dentists - Followed a structured questionnaire - Two 6-point scale response question to evaluate intervention effectiveness | ***Clinical***   - NNT for de was lower for Group A (4) than for Group B (9). - No significant difference in dt. - No new de appeared in group A, the mean de increment in group B was 0.2 (0.6), and 0.4 (0.7) in controls (*p*<0.05). - The percentages of children developing new de were 0% (Group A), 14% (Group B) and 26% (Controls).   ***Mother’s perception of the effectiveness of the intervention***   - 88% of mothers were ‘very much’ or ‘much’ satisfied with pamphlet, 64% perceived ‘much’ or ‘very much’ change in oral health behaviours in both intervention groups. - Group A thought pamphlet had more influence on changing OH behaviours compared to Group B (*p*=0.05).   Oral health education provided by non-dental professionals working in general health settings is a feasible way of preventing or slowing caries increments in early childhood in countries with developing oral health systems. | 2 |
| **Hallas et al. (2015) (**[**3**](#_ENREF_34)**5)**  **United States** | **Prospective randomised controlled trial**  Determine the oral health hygiene knowledge of mothers of newborns and the effectiveness of an oral health education program on the oral health of infants at 6 and 12mo. | 94 mother-baby dyads  ***Intervention:*** n=47  ***Comparison:*** n=47  ***Mothers of newborn babies***   - 48% spoke both English & native language - 52% spoke Spanish-only - 60% were Latina | Nurses/midwives* | ***Intervention group***  Oral health education - DVD   - 8 min DVD on oral health care of newborns through 6mo   ***Control group***  8 min DVD on nutrition for newborns and infants  ***All groups***   - Received routine newborn nursery education by nurses, lactation consultant, physician and residents. - Oral hygiene kit for newborn - Newborn oral health brochure - Telephone and mail contact at 6mo and 12mo to remind appointment date - Oral and written (brochure) instructions on infant oral health care to prevent caries (distributed after each appointment at 6mo and 12mo). | ***Clinical - dental health status***   - Infant oral health status scored 0, 1, 2 based on “no cavitation”, “white spots” and “cavitation”, respectively - Performed by dental professional and/or paediatric nurse practitioner (PNP) student at 6mo and 12mo post-partum.   ***Clinical – risk of ECC***   - Six history items scored to determine the child’s overall risk of caries based on oral intake and history of dental caries in child and parent.   ***Behaviour – knowledge and practices***   - 7-item pre/post questionnaire. - Assessed mother’s oral health knowledge, beliefs and practices. - Administered at baseline and 6mo. | ***Clinical***   - Oral health assessment at 6 and 12 mo identified no white spots and all infants (n=10) were cavity free. - Infants who returned for follow-up at 6mo & 12mo (n=10) were at high risk of ECC.   ***Behaviour***   - Baseline results indicated mother’s lack of oral health knowledge about infants and young children, especially relating to the effects of vertical transmission of *streptococcus mutans* from mother to baby.   Note: Significant no-show rate for the planned 6 and 12mo follow-ups hindered the evaluation of the program on prevention of dental white spots or decay. | 3 |
| **BOTH ANTENATAL/POSTNATAL PERIODS** | | | | | | | |
| **Milgrom et al. (2010) (**[**3**](#_ENREF_35)**8)**  **United States** | **Quasi-experimental with a comparison group**  To determine the benefits accrued to the offspring of mothers who participated in a community-based public health program providing dental home in a rural area | 235 mother-child dyads  ***Intervention:*** n=235  ***Comparison***: n=56  ***Mothers***  Income and education characteristics of caregivers poorly defined  ***Children***  Intervention   - Mean age 24mo - 50% males - 16% Hispanic - 47.3% first child   Comparison  Comparable children (24-35mo) from non-program counties   - Mean age 28mo - 55% males - 56% Hispanic - 47.2% first child | Outreach coordinator (OHSC)*   - Health department employee - Provided home visits | ***Intervention group***  Oral health education – Home visits   - OHSC provided information on the mother seeing the dentist and oral health practices. - Distributed oral health toolkits (English/Spanish).   Oral health education – Counselling sessions   - Mothers attended counselling sessions at the Women, Infant, and Children (WIC) program of the county health department.   Dental referral   - Mothers were assigned a dental home under a dental managed care program. - Received dental care during pregnancy or within 2mo of delivery.   ***Control group***  Did not participate in program. Received usual care. | ***Clinical - dental health status***   - Number of children with any decayed deciduous tooth and number of decayed teeth. - Visual tactile examination using the WHO criteria (only frank cavitation was recorded as tooth decay). - Conducted by the dental managed care organizations in dental offices. - Measured at child’s 2^nd^ year of life. | ***Clinical***   - Overall 85% of the children aged 24-28 mo in the intervention group were caries free compared to 58.9% of controls (RR = 1.48, 95% CI 1.13 -1.93). Therefore, children in the intervention group were almost 1.5 times more likely to be caries free than children in the comparison counties. - There was a statistically significant lower mean (SD) for number of teeth with any decay in the intervention group 0.75 (2.5) compared to the comparison group 1.6 (2.5) (t = 2.08, *p* = 0.04). | 3 |
| **Chaffee et al. (2013) (**[**2**](#_ENREF_25)**7)**  **Brazil** | **Cluster randomised controlled trial**  To estimate the caries impact of providing training in infant feeding guidelines to workers at Brazilian public primary care clinics. | 458 mother-child dyads  20 Clinics  ***Intervention:*** n=9  ***Comparison:*** n=11  ***Mothers***   - Mean age at infant delivery 26.4y - 55.75% white - 53.35% “Never” smoked during pregnancy - 99.2% literate - 46.7% ≤8y of formal education, - $1085 -household monthly income - 4.2 household members on average   ***Children***   - Male child (50.9%) | Healthcare workers   - Physicians - Nurses - Administrative staff - 1hr training by a nutritionist to incorporate into maternal consultations | ***Intervention group***  Oral health education – Counselling   - Dietary counselling for pregnant/lactating women aimed to improve oral health outcomes. - Posters displayed at intervention clinics. - Pamphlets distributed to pregnant/lactating women. - Messages were relayed to mothers with unknown consistency and accuracy.   **Control group**  Usual practice which allowed for maternal counselling at practitioner discretion. | ***Clinical - dental health status***   - Tooth surfaces were recorded as “sound”, “decayed non-cavitated”, “cavitated”, missing due to caries, or restored (WHO protocol) - Visual dental assessment (2-3y) - Conducted by dentist at participant’s home   ***Behaviour – Service uptake***   - Mothers were asked by trained field workers at 2-3y whether their child had ever visited a dentist   ***Mother’s experiences of intervention***   - Trained field workers asked at 5-9mo & 11-15mo whether they: - Attended same health centre (as enrolment) - Viewed Ten Steps poster - Received Ten Steps pamphlet | ***Clinical***   - Reduction in ECC (52.3% vs 57%), cavitated decay (37.1% vs 42.1%), or S-ECC (32.1% vs 36.7%) was not significantly different between the intervention and control groups. - Health care workers training in infant feeding practice and nutrition did not yield a statistically significant reduction in caries overall.   ***Mother’s experiences of intervention***   - There was a statistically significant reduction in S-ECC among mothers more connected to their health centres (i.e. who remained exclusively at the same health centre (RR 0.68; 95% CI, 0.47-0.99) and among mothers who named the health centre as their principal source of feeding advice (RR 0.53; 95% CI, 0.29-0.97.   ***Behaviour***   - 26.6% reported previously visiting a dentist, and this was not significantly different between the 2 groups. | 1 |

*: Trainer not specified; CI: confidence interval; CSA: Central Services Agency, an organisation responsible for providing support services to health and social work agencies in Ireland; De: enamel caries; DS or ds: dental surface; Dt: decayed teeth; ECC: early childhood caries; GDPs: General Dental Practitioner; HE: health education; Hr: hour(s); MI: motivational interviewing; Min: minute(s); mo: month(s); N/S: Not specified; SD: standard deviation; S-ECC: Severe early childhood caries; T1/T2: Time-point 1/Time-point 2; Wks: weeks; Y: year(s)
