# Supplementary material for: Effectiveness of preventive dental programs offered to mothers by non-dental professionals to control early childhood dental caries: a review
Source: BMC Oral Health. 2019 Aug 2;19:172. doi: 10.1186/s12903-019-0862-x (PMC6679429; doi:10.1186/s12903-019-0862-x)
Supplement: Supplementary file 3 — Quality Assessment of Included Studies. Results of quality assessment using NIH tool. (DOCX 13 kb) [file 12903_2019_862_MOESM3_ESM.docx]

Additional File 3 Quality Assessment of Included Studies

| **Author** | **National Heart, Lung, and Blood Institute (NIH) Quality Assessment of Studies - Items** | | | | | | | | | | | | | | | | |
| --- | --- | --- | --- | --- | --- | --- | --- | --- | --- | --- | --- | --- | --- | --- | --- | --- | --- |
|  | **1** | **2** | **3** | **4** | **5** | **6** | **7** | **8** | **9** | **10** | **11** | **12** | **13** | **14** | **Total (/14)** | **Quality Assessment Score** |  |
| Chaffee et al. (2013) | Y | Y | Y | Y | N | Y | N | Y | Y | Y | Y | N | Y | Y | 11 | 2 |  |
| Feldens et al. (2007) | Y | Y | N | N | Y | Y | Y | Y | Y | Y | Y | Y | Y | Y | 12 | 1 |  |
| Hallas et al. (2015) | Y | N | N | N | N | N | N | N | Y | Y | Y | Y | N | N | 5 | 3 |  |
| Harrison et al. (2003) | N | N | N | N | N | N | Y | N | Y | Y | Y | N | N | N | 4 | 3 |  |
| Larsen et al. (2016) | Y | Y | Y | Y | N | N | Y | N | Y | Y | N | N | N | N | 7 | 3 |  |
| Milgrom et al. (2010) | N | N | N | N | N | Y | N | N | Y | Y | Y | N | N | N | 4 | 3 |  |
| Mohebbi et al. (2009) | Y | Y | Y | Y | Y | Y | N | Y | Y | N | Y | Y | N | N | 10 | 2 |  |
| Weinstein et al. (2004) | Y | Y | N | N | N | Y | Y | N | Y | Y | Y | N | N | N | 7 | 3 |  |
| Yuan et al. (2007) | N | N | N | N | N | Y | N | N | Y | Y | N | N | Y | N | 4 | 3 |  |
